# A Retrospective Study of the Efficacy of Albendazole and Diethylcarbamazine for the Treatment of Human Toxocariasis

**DOI:** 10.3390/pathogens11070813

**Published:** 2022-07-20

**Authors:** Jean-François Magnaval, Judith Fillaux, Antoine Berry

**Affiliations:** 1 Service de Parasitologie Médicale, Faculté de Médecine, Université de Toulouse III, 37 allées Jules-Guesde, 31000 Toulouse, France; 2 Service de Parasitologie-Mycologie, Hôpital Purpan, Centre Hospitalier Universitaire de Toulouse, 330 avenue de Grande-Bretagne, 31059 Toulouse, France; fillaux.j@chu-toulouse.fr (J.F.); berry.a@chu-toulouse.fr (A.B.); 3 UMR Infinity, Institut Toulousain des Maladies Infectieuses et Inflammatoires, Inserm-CNRS-Université de Toulouse III, 31024 Toulouse, France

**Keywords:** toxocariasis, treatment, observational study, retrospective study, albendazole, diethylcarbamazine

## Abstract

In the Department of Parasitology and Mycology of Toulouse University Hospitals, patients presenting with common/covert toxocariasis were treated either with albendazole (39 cases) or with diethylcarbamazine (32 cases). Albendazole (ABZ) was given at 10 mg/kg b/w daily for 14 days, and diethylcarbamazine (DEC) was given at 4 mg/kg b/w daily for 21 days. In both groups, follow-up consultations occurred approximately 48 days after the end of the anthelmintic therapy. ABZ and DEC displayed a similar efficacy on the kinetics of the clinical picture (−64.5% of reduction vs. −72.7%, respectively) and on the levels of blood eosinophilia, serum eosinophil cationic protein and serum total IgE. However, the effect of the medication on the laboratory parameters was moderate. The rate of adverse reactions was similar in both groups (38% for ABZ vs. 31% for DEC), but DEC-treated patients complained of more intense and long-lasting side effects. The DEC group had more major adverse reactions, resulting in the termination of the anthelmintic treatment. The results from this retrospective study bring further arguments for considering ABZ, given at 10 mg/kg daily for 2 weeks, as the drug of choice in the treatment of human toxocariasis.

## 1. Introduction

Human toxocariasis is a worldwide zoonotic helminthiasis caused by infection with the larvae of *Toxocara canis* or *Toxocara cati*. Both species parasitize the upper digestive tract of canids or felids [1]. Eggs that are passed in the feces must be in the soil to become embryonated and infectious. Most frequently, humans become infected by ingesting embryonated eggs that are present in nearby soil or on raw vegetables [2,3]. *Toxocara* infection can elicit several disorders that can be classified as either systemic (generalized)—including major visceral larva migrans (VLM) syndrome and covert/common toxocariasis—or compartmentalized when the eye or the central nervous system are involved [1]. Various allergic signs or symptoms are commonly observed during the course of systemic syndromes [4].

To date, there is no standardized anthelmintic therapy for human toxocariasis. Only four compounds are available for human use, namely, albendazole (ABZ), diethylcarbamazine (DEC), mebendazole (MBZ) and thiabendazole (TBZ). Their efficacy has rarely been assessed in prospective controlled trials. ABZ, which is considered the drug of choice due to its quasi-worldwide availability, low price, lack of major adverse reactions and seemingly acceptable efficacy, has been the subject of only one randomized two-arm trial [5]. The other six studies were observational, prospective or retrospective [6].

As of 1984 [7], investigations concerning human toxocariasis and the management of *Toxocara*-infected patients have been a prominent research axis in the Department of Parasitology and Mycology in Toulouse University Hospitals. The aim of the present retrospective, observational study was to assess the efficacy and tolerance of two anthelmintic regimens using ABZ or DEC for the treatment of common/covert toxocariasis.

## 2. Patients and Methods

### 2.1. Diagnosis of Common/Covert Toxocariasis

At the Outpatient Clinic of the Department of Parasitology and Mycology in the Toulouse University Hospitals, France, the diagnosis of active common/covert toxocaral disease was made at the end of a meticulous protocol that has been detailed elsewhere [8]. It should be reiterated that the clinical and laboratory picture of the most frequent form of this zoonotic disease is not specific [1,7]. Moreover, many subjects had residual anti-*Toxocara* antibodies due to past self-resolved infections. Therefore, the diagnosis of active common/covert toxocariasis was made by exclusion in patients who exhibited a positive result for toxocariasis serology once the aforementioned protocol ruled out other causes of eosinophilia. All patients were investigated by the same author (JFM).

For any patient who was diagnosed as having only common/covert toxocariasis, they were given a detailed questionnaire that inquired about demographics (age and sex) and environmental data and was recorded along with the patient’s medical history. The time interval between the onset of manifestations and attendance at the clinic was recorded in months. The clinical picture was evaluated based on the general examination of the patients and was quantified using a rating procedure: any sign or symptom that was consistent with the presence of common/covert toxocariasis was rated as 2 if it was present and 0 if was absent. At the follow-up consultation, the rating was 3 if an increase was noted, 2 if the expression of the parameter was stable, 1 if it had reduced and 0 if it had vanished. Plain chest radiography and abdominal ultrasound were also performed. Ophthalmological examinations were also required to rule out any ocular involvement consistent with ocular toxocariasis.

### 2.2. Laboratory Methods

The immunodiagnosis of toxocariasis was based upon a Western-blotting procedure (WB) that detected specific IgG against *Toxocara canis* excretory–secretory larval antigens. This antigenic reagent was produced in the Department of Parasitology and Mycology. The presence of a banding pattern that displayed positive reaction for the lower molecular weight bands (24, 28, 30, and 35 kDa) was evidence of a positive result [9].

Total and differential blood counts were performed routinely in the Department of Haematology on various autoanalyzers, and assays for serum total IgE and eosinophil cationic protein (ECP) were routinely carried out in the Department of Immunology.

### 2.3. Drugs and Treatment Policies

In the mid-1990s, a retrospective analysis was carried out using the file records of the patients who had attended the clinic since 1982 [unpublished data]. The results indicated that common/covert toxocariasis was mostly a self-limiting disease that did not require any anthelmintic therapy provided appropriate measures preventing reinfections were implemented. Consequently, it was decided that only clinically symptomatic patients presenting with long-lasting disease had to be treated. It was estimated that an approximately 3-month interval of time between the onset of the disease and the attendance at the clinic was the criterion supporting the decision to give the patient an anthelmintic treatment.

The availability of anthelmintics for human toxocariasis has varied over time in France due to successive changes in the legal regulations of these drugs. Since the results emerged of a study comparing DEC and MBZ, the latter has been the first choice in our department because it has been found to be as efficient as DEC but elicits a significantly lower rate of side effects [10]. By the 2000s, MBZ had become only available through a long administrative procedure of drug importation. ABZ has not been used because its possible drug regimens for toxocariasis were outside the field of French approval. That is, by that time, the upper limit of the approved regimen for treating strongyloidiasis was 400 mg/day for three days. Consequently, all patients presenting with common/covert toxocariasis were treated with DEC. In the mid-2000s, ABZ received an extended approval for the treatment of trichinellosis using a dosage of 10 mg to 15 mg b/w daily for 10 to 15 days. This daily dosage, along with longer regimens, was also approved for the medical treatment of larval echinococcosis. By extension, using this protocol for the treatment of toxocariasis was legally admissible, so ABZ became our drug of choice.

When DEC was used, the regimen was 4 mg/kg b/w daily for 21 days. The therapeutic schedule started at 25 mg daily, and the dose was progressively increased to avoid adverse reactions due to larval lysis. No antihistamine drugs were used. For ABZ, the daily dose was 10 mg/kg b/w not to exceed 800 mg daily and the duration of therapy was 14 days. Regardless of the anthelmintics that were used, the treated patients were required to attend the clinic for a follow-up consultation one month after their treatment had ended. They were advised that this time interval could be increased but would not be shortened. The choice of only one follow-up examination was due to practical considerations. Attending an outpatient clinic could be a complicated affair for certain patients who sometimes had to travel more than 100 km. Previous experience showed that patients often did not show up for second follow-up consultations, so we decided that the continuation of follow-up would be ensured with simply a direct collaboration with the patients’ personal physicians.

In both groups, the patients were informed at their first consultation about measures that can prevent reinfection and were urged to implement these measures [8].

### 2.4. Criteria for Retrospective Inclusion in the Study and Statistical Analysis

Cases of ocular or neurological toxocariasis were excluded. Additionally, the only patient with VLM was excluded because he received both anthelmintics and corticosteroids. Therefore, only the file records of the patients who had presented with common/covert toxocariasis and who had fulfilled the aforementioned treatment policies were considered. Moreover, patients had to complete the drug therapy and attend the follow-up consultation. According to these criteria, 71 file records were extracted from the period between 1996 and 2012. A total of 39 patients were treated with ABZ, and 32 were treated with DEC, and the data were anonymized before statistical analysis.

To assess the efficacy of ABZ and DEC, a bivariate analysis of the continuous and categorical variables was carried out. The continuous clinical variables included the clinical score of the disease, which was determined by the aforementioned rating procedure, and the laboratory parameters, including the blood eosinophil count, eosinophil cationic protein (ECP) and serum total IgE levels. Categorical variables were generated at the follow-up by classifying the results according to a cutoff value. For the clinical score, this value was defined as a 75% decrease, namely, an approximate 1.5-fold decrease greater than the placebo effect that is usually observed in this kind of study [11]. After considering the results of the eosinophil count, titration of ECP and total serum IgE, a positive effect post-anthelmintic therapy was defined as a return to normality.

The statistical package Intercooled Stata™ (StataCorp LLC, College Station, TX, USA) along with Epi-Info (Center for Diseases Control, Atlanta, GA, USA) were used for the statistical analysis. Bivariate analysis of the data set used Mantel–Haenszel χ^2^ and Fisher’s tests as appropriate for the categorical variables or the Mann–Whitney *U* and Wilcoxon signed-rank tests as appropriate for the continuous variables. Since the Mann–Whitney and Wilcoxon tests are nonparametric, only the interquartile range values and not the standard deviation values are displayed in the tables.

## 3. Results

The demographic characteristics and environmental features of the patients treated either with ABZ or DEC are displayed in Table 1, along with the results of the bivariate analysis of the collected variables.

The statistical analysis showed that the ABZ and DEC groups were similar, particularly in age and sex. The significant difference in the status of pet cats, namely, dewormed or not, likely expresses the evolution of the public knowledge about proper pet care and the prevention of human toxocariasis. As of 1993, *T. cati* had been suggested as another agent of human toxocariasis [12]. Consequently, by the mid-1995s, cat deworming was added to the recommended preventive measures for all toxocariasis patients.

Table 2 displays the most prominent clinical signs or symptoms that were observed by the personal physicians and that prompted the physicians to refer these 71 patients to the clinic. Additionally, the table describes the components of the clinical picture that were noticed at the first consultation. The mean duration of the disease before consultation, namely the interval between the onset of the disease and attendance at the clinic, is reported as well. The analysis of the results from both plain chest radiography and abdominal ultrasound showed no abnormalities that would have been related to toxocariasis.

Four patients in the DEC group who had been determined to be asymptomatic by their personal physicians were referred to the clinic due to the finding of a chronic blood eosinophilia. However, upon examination, these patients exhibited certain clinical issues as shown in Table 2. The bivariate analysis of the clinical variables showed that only the presence of skin allergy issues, which had initiated the consultation or were found on the consultation examination, was significantly different between the ABZ and DEC groups.

Table 3 shows the comparison of the continuous variables before treatment and at the follow-up step.

The dichotomization of the collected clinical and laboratory data provided results that were consistent with the analysis of the continuous variables (Table 4).

The nature and rate of adverse reactions (ARs) are presented in Table 5. Six patients who were treated according to the aforementioned policies were not included in the study. These persons had to discontinue anthelmintic therapy due to the development of major Ars, and these ARS are described at the end of Table 5.

## 4. Discussion

Since the present study was not prospective and randomized but rather was observational and retrospective, any comparison between the two groups of patients could be questionable. However, the implementation of a constant and rigorous treatment policy led to the ABZ and DEC groups being very similar. In particular, the bivariate analysis of the demographic characteristics and environmental factors that were possible confounding variables showed that the ABZ and DEC groups were similar. The only significant difference that was found was a greater proportion of not-dewormed cats in the DEC group. However, this could not affect the drug efficacy since these animals had to be dewormed following the patient’s first consultation.

A significantly greater proportion of patients presenting with skin allergy signs was observed in the ABZ group (Table 2). One explanation for this could be the increasing awareness of the full clinical spectrum of toxocariasis by general practitioners and specialized doctors. *Toxocara* infection was shown to be an etiology of chronic urticaria in the mid-1990s [13], and this was then confirmed in subsequent studies [14,15]. Consequently, this information was added to the nosography of human toxocariasis in our medical teaching and was also delivered to practicing doctors through postuniversity educational sessions.

The patients’ follow-up consultations occurred between 40 and 80 days after the end of anthelmintic therapy (Table 3). ABZ and DEC demonstrated a significantly similar efficacy based upon the kinetics of the quantified clinical picture and the assessed laboratory assays. According to the data that were obtained from a review of the literature [6], it could be objected that follow-up was too close to the end of both treatments. As indicated in Section 2.3, the follow-up continuation was with the patients’ personal physicians. The doctors were asked to check the evolution of the residual clinical signs or symptoms and the eosinophil count as well. This check-up had to take place three months from the date of the follow-up consultation at the clinic but could not occur prior to this. Six (15.4%) patients in the ABZ group and four (12.5%) in the DEC group were referred to the clinic again, which was on average 99.9 ± 108.5 days after the first follow-up consultation. The differences in the distribution (Fisher’s exact test) were not significant.

This procedure provided circumstantial evidence that two anthelmintic therapies had similar efficacies, ranging from 74.6% for ABZ to 87.5% for DEC. For ABZ, a Korean study reported a similar 85.1% efficacy 6 months after therapy using an 800 mg daily dose [16] The use of continuous (Table 3) and categorical (Table 4) variables showed similar kinetics in assessing the clinical efficacy of both anthelmintics.

The two groups showed similar rates of ARs (Table 5). Upon interrogation during the follow-up consultation, most events were coined mild by the ABZ-treated patients, whereas they were often depicted as more unpleasant and long-lasting in the DEC group. Pruriginous rashes, which were observed at the very early stage of ABZ or DEC therapy, were evocative of fast larval lysis, similar to the well-known Mazzotti’s reaction. This AR was primarily identified during the treatment of onchocerciasis with DEC, [17] but it also occurred in patients with tissue-dwelling helminthiases [18,19]. Major ARs that resulted in the discontinuation of the therapy were mostly recorded following DEC use (Table 5). The intensity and nature of ABZ-induced ARs did not differ from the results obtained from the published trials that used very similar drug regimens [6].

Concerning DEC, only one prospective randomized trial inquired about DEC use [10]. As in the present study, DEC was given at 3–4 mg/kg b/w daily for 21 days in common/covert toxocariasis patients. This drug regimen was lower than the standard for filariases, which is 6 mg/kg b/w for 21 days. The choice of a reduced daily dose relied upon differences in the pathophysiology between filariases and human *Toxocara* infection. That is, the whole larval load in filariases such as loiasis or onchocerciasis is obviously by far higher than that in common/covert toxocariasis. In 1959, a human volunteer was given approximately 100 *T. canis* embryonated eggs orally. His blood eosinophil count increased to 13,500 cells/mm^3^ on day 30 postinfection and remained at 6150 cells/mm^3^ at 4.5 months later. This volunteer also developed a persistent cough [20]. Between the two DEC trials, the results concerning the efficacy, clinical picture and blood eosinophilia appeared to be similar. Again, DEC therapy was found to not affect the total IgE kinetics. Finally, the rate of ARs did not significantly differ between the two studies.

Concerning ABZ, it was not possible to obtain an accurate comparison with other trials involving this drug due to the great variations in the targeted forms of toxocariasis (VLM vs. common/covert toxocariasis) and in the treatment schedules between the studies. Regardless, decisive trends have emerged after 7 major trials which were reviewed [6]. First, it appears to be crucial to rule out patients whose disease will self-resolve [16]. In the present study, the average duration of toxocariasis before consultation was over one year (Table 2). Combined with the fast posttreatment decrease in both the mean clinical score and mean eosinophil count, this finding suggested that the pitfall of a self-cure was avoided in the present study. Moreover, the drug regimens should include a sufficient daily dose and an appropriate duration of the treatment. In these 7 trials, the daily dose for ABZ ranged from 10 to 15 mg/kg b/w and did not exceed 800 mg/day. The duration of the treatment varied between 5 and 56 days. When compared with the most commonly used 2-week schedule, the longest treatment regimens did not result in a sharp increase in the cure rate.

## 5. Conclusions

In conclusion, this retrospective study supports the use of ABZ given at a 10–15 mg/kg b/w daily dose for a 2-week course as the treatment of choice for human toxocariasis. This regimen will avoid the major side effects that have been reported with the long-term regimens, e.g., in the treatment of larval echinococcosis [21]. The quantification of the clinical picture appears to be useful for prospective studies but not necessary for individual assessments. Although it has a similar global efficacy to ABZ, DEC should only be considered as an alternative option, and it should be restricted to patients who have failed ABZ therapy.

## Figures and Tables

**Table 1:** Demographic and environmental characteristics of 71 common/covert toxocariasis patients treated with ABZ (*n* = 39) or DEC (*n* = 32).

	ABZ ^1^ (*n* = 39)	DEC ^2^ (*n* = 32)	*p*
*Age (years)*			
Mean (IQR ^5^)	50.05 (27)	44.94 (24)	NS ^3,4^
*Sex % (n)*			
Females	64.1 (25)	50 (16)	NS ^3,6^
Males	35.9 (14)	50 (16)	
*Weight (kg)*			
Mean (IQR ^5^)	69.74 (19)	68.62 (22)	NS ^3,4^
*Residentship % (n)*			
Rural and towns ≤ 5000 inhabitants	53.9 (21)	68.75 (22)	NS ^3,6^
Towns ≥ 5000 inhabitants	46.1 (18)	31.25 (10)	
*Number of pet dogs*			
Mean (IQR ^5^)	2.2 (2)	2.2 (3)	NS ^3,4^
*Dogs’ status per household % (n)*			
Dewormed	39.8 (12)	18.75 (6)	NS ^3,6^
Not dewormed	69.2 (27)	81.25 (26)	
*Number of pet cats*			
Mean (IQR ^5^)	1.2 (2)	0.75 (0)	NS ^3,4^
*Cats’ status per household % (n)*			
Dewormed	20.5 (8)	0 (0)	0.0058 ^7^
Not dewormed	79.5 (31)	100 (32)	
*Personal kitchen garden % (n)*			
Yes	59 (23)	59.4 (19)	NS ^3,6^
No	41 (16)	40.6 (13)	

^1^: albendazole; ^2^: diethylcarbamazine; ^3^: not significant; ^4^: Mann-Whitney U test; ^5^: interquartile range; ^6^: Mantel-Haenszel χ^2^; ^7^: Fisher’s exact test.

**Table 2:** Clinical features of common/covert toxocariasis in 71 patients treated with ABZ (*n* = 39) or DEC (*n* = 32).

	ABZ ^1^ (*n* = 39)	DEC ^2^ (*n* = 32)	*p*
*Interval (months) between the onset of the disease and presentation to the Outpatient Clinic*			
Mean (IQR ^3^)	13.90 (8)	16.66 (20)	NS ^4,5^
*Clinical reason for presenting to the Outpatient Clinic % (n)*			
Arthralgia and/or myalgia	5.2 (2)	0 (0)	NS ^4,6^
Asymptomatic ^7^	12.5 (4)	0 (0)	0.039 ^6^
Chronic weakness	43.6 (17)	59.4 (19)	NS ^4,8^
Chronic irritative cough	2.6 (1)	6.25 (2)	NS ^4,6^
Facial and/or hand edema	2.6 (1)	0 (0)	NS ^4,6^
Febrile lung involvement	2.6 (1)	0 (0)	NS ^3,6^
Fever	0 (0)	3.1 (1)	NS ^4,6^
Gastric pain	0 (0)	6.25 (2)	NS ^4,6^
Intermittent diarrhea	2.6 (1)	0 (0)	NS ^3,6^
Paresthesia	0 (0)	3.1 (1)	NS ^4,6^
Skin allergy ^9^	28.2 (11)	2.6 (1)	0.0086 ^6^
Weight loss	2.6 (1)	0 (0)	NS ^4,6^
Wheezes	10.3 (4)	6.25 (2)	NS ^4,6^
*Clinical data recorded at the first consultation % (n)*			
Arthralgia and/or myalgia	33.3 (13)	40.6 (13)	NS ^4,8^
Chronic irritative cough	23.1 (9)	18.8 (6)	NS ^4,8^
Chronic weakness	76.9 (30)	87.5 (28)	NS ^4,8^
Colic pain	28.2 (11)	28.2 (9)	NS ^4,8^
Conjunctivitis	30.8 (12)	15.6 (5)	NS ^4,8^
Facial and/or hand edema	10.3 (4)	12.5 (4)	NS ^4,6^
Frequent headache	20.5 (8)	18.8 (6)	NS ^4,8^
Intermittent diarrhea	2.6 (1)	9.4 (3)	NS ^4,6^
Otorhinolaryngeal allergy ^10^	82.05 (32)	75 (24)	NS ^4,8^
Paresthesia	7.7 (3)	3.1 (1)	NS ^4,6^
Pruritus sine materia	33.3 (13)	18.8 (6)	NS ^4,8^
Shortness of breath	2.6 (1)	9.4 (3)	NS ^4,6^
Skin allergy ^9^	38.5 (15)	12.5 (4)	0.017 ^6^
Wheezes	7.7 (3)	9.4 (3)	NS ^4,6^

^1^: albendazole; ^2^: diethylcarbamazine; ^3^: interquartile range; ^4^: not significant; ^5^: Mann-Whitney *U* test; ^6^: Fisher’s exact test; ^7^: only blood eosinophilia; ^8^: Mantel-Haenszel χ^2^; ^9^: eczema, pruriginous rashes, urticaria; ^10^: pharyngitis, rhinitis (rhinorrhea, nasal congestion, sneezing), sinusitis.

**Table 3:** Bivariate analysis of the continuous variables recorded from 71 patients treated with ABZ (*n* = 39) or DEC (*n* = 32).

		Mean (IQR) ^1^		
*Interval (days)* ^2^				
ABZ ^3^	48.05 (18)			
*p* ^4^	NS ^5^			
DEC ^6^	47.9 (16)			
	Before treatment	After treatment	*p* ^7^	% variation
*Clinical score*				
ABZ ^3^	7.95 (8)	2.82 (3)	<0.00001	−64.45 (33)
*p* ^4^	NS ^5^	NS ^5^		NS ^5^
DEC ^6^	7.25 (6)	2.28 (3)	<0.00001	−72.68 (50)
*Eosinophil count (cells G/L)*				
ABZ ^3^	1.4 (0.8)	0.69 (0.5)	<0.0001	−37.7 (54.8)
*p* ^4^	NS ^5^	NS ^5^		NS ^5^
DEC ^6^	1.23 (1.18)	0.84 (0.84)	0.00044	−18.8 (42.3)
*ECP ^8^ (µg/L)*				
ABZ ^3^	54.1 (65)	27 (29)	<0.0001	−28.5 (56.8)
*p* ^4^	NS ^5^	NS ^5^		NS ^5^
DEC ^6^	40.4 (37)	29 (22)	0.0271	−5.5 (76.7)
*Serum total IgE ^9^ (kIU/L)*				
ABZ ^3^	1442 (1333)	1167 (904)	0.00244	−9.4 (34.2)
*p* ^4^	NS ^5^	NS ^5^		NS ^5^
DEC ^6^	1462 (1660)	1390 (1252)	NS ^7^	−4.6 (39.4)

^1^: interquartile range; ^2^: interval between the end of therapy and the follow-up consultation; ^3^: albendazole; ^4^: Mann-Whitney *U* test; ^5^: not significant; ^6^: diethylcarbamazine; ^7^: Wilcoxon’s signed rank test; ^8^: eosinophil cationic protein; normal values ≤ 14 µg/L; ^9^: normal values ≤ 150 kilo International Units/L.

**Table 4:** Bivariate analysis of the categorical variables recorded from 71 patients treated with ABZ (*n* = 39) or DEC (*n* = 32).

	ABZ ^1^	DEC ^2^	*p*
	% (*n*)	% (*n*)	
Decrease in the clinical score ≥ 75%	46.15 (18)	56.25 (18)	NS ^3,4^
Eosinophil count ≤ 0.5 G/L	33.3 (13)	28.12 (9)	NS ^3,4^
Eosinophil cationic protein ≤ 14 µg/L	23.7 (9)	28.12 (9)	NS ^3,4^
Serum total IgE ≤ 150 kIU/L ^5^	2.6 (1)	0 (0)	NS ^3,6^

^1^: albendazole; ^2^: diethylcarbamazine; ^3^: not significant; ^4^: Mantel-Haenszel χ^2; 5^: kilo International Units; ^6^: Fisher’s exact test.

**Table 5:** The adverse reactions observed in 71 patients treated with ABZ (*n* = 39) or DEC (*n* = 32).

ABZ ^1^		DEC ^2^	
	% (*n*)		% (*n*)
Patients with AR ^3^	38.5 (15)	Patients with AR ^3^	31.25 (10)
NS ^4 5^			
Colic pain	1 (case)	Colic pain	1 (case)
Dizziness	2	Dizziness	2
Drowsiness	1	Drowsiness	1
Insomnia	1	Gastric pain	3
Loose stools	1	Headache	1
Loss of appetite	1	Nausea	4
Nausea	3	Pruriginous rash ^6^	2
Pruriginous rash ^6^	2	Vomiting	1
Weakness (intense)	3	Weakness (moderate)	2
Weakness (moderate)	2		
Additional data ^7^			
Patients with common/covert toxocariasis who discontinued anthelmintic treatment			
(*n* = 6)			
Intense vomiting	1 (case)	Major asthma attack	1 (case)
		Intense vomiting	1
		Acute dizziness	1
		Acute confusion and vertigo ^8^	1
		Generalized pruriginous rash ^6^	1

^1^: albendazole; ^2^: diethylcarbamazine; ^3^: adverse reactions; several might occur simultaneously in a given patient; ^4^: not significant; ^5^: Mantel-Haenszel χ^2^; ^6^: likely due to larval lysis/Mazzotti-like reaction; ^7^: not included in the study; ^8^: requiring emergency hospitalization.

## Data Availability

The data presented in this study are available on request from the corresponding author. The data are not publicly available due to legal restrictions.
